# Localized bigeminal activity in the left atrial posterior wall preceding atrial fibrillation initiation

**DOI:** 10.1016/j.jccase.2026.06.008

**Published:** 2026-07-15

**Authors:** Ko Abe, Kazuhiro Nagaoka, Yasushi Mukai, Hiroshi Kojima, Shunji Hayashidani, Hideki Tashiro

**Affiliations:** a Department of Cardiovascular Medicine, St. Mary's Hospital, Kurume, Japan; b Department of Cardiovascular Medicine, Japanese Red Cross Fukuoka Hospital, Fukuoka, Japan

**Keywords:** Atrial fibrillation, Bigeminy, Non-pulmonary vein, Posterior wall of left atrium, Vein of Marshall

## Abstract

Bigeminal activity of the pulmonary veins is considered an indicator of arrhythmogenic triggers of atrial fibrillation (AF). However, few reports have described localized bigeminal activity originating from non-pulmonary vein regions. We observed the phenomenon of local bigeminal activities, which did not develop into premature atrial contraction, in a restricted area of left atrial posterior wall (LAPW), preceding the initiation of the AF. Radiofrequency ablation targeting the site of these bigeminal activities transiently suppressed AF, and subsequent linear ablation connecting the site to the adjacent low-voltage region resulted in durable elimination of AF. The spatial relationship between the localized activity and the low-voltage substrate suggests that conduction abnormalities within diseased atrial myocardium may have contributed to the observed phenomenon. This case highlights the potential role of localized electrical activity arising near a low-voltage substrate of the LAPW in AF initiation and may provide insights into the electrophysiological mechanisms of non-pulmonary vein triggers.

**Learning objective:**

Left atrial posterior wall (LAPW) is known to be one of the non-pulmonary vein (PV) origins triggering atrial fibrillation (AF). While bigeminal activity originating from PVs or superior vena cava has been associated with arrhythmogenic triggers, similar activity originating from non-PV origin, such as LAPW, has rarely been described. This case indicates that localized bigeminal activity occurring near a low-voltage substrate of the LAPW may precede AF initiation and provides new insights into non-PV arrhythmogenic substrates as the target of ablation.

## Introduction

The pulmonary veins (PVs) are well recognized as major sources of atrial fibrillation (AF) initiation [1]. Nevertheless, despite durable and complete pulmonary vein isolation (PVI), AF recurs in some populations, highlighting the clinical importance of additional arrhythmogenic substrates such as non-pulmonary vein (non-PV) triggers [2], [3]. Bigeminal activity originating from the PVs has been identified as an indicator of arrhythmogenic activity triggering AF. Such activity has been suggested to reflect abnormal conduction properties or localized reentrant circuits within the PV musculature and/or the surrounding atrial tissue [4]. Similarly, bigeminal activity from the superior vena cava (SVC), one of the well-established targets for non-PV origins, may also be associated with potent arrhythmogenicity [5]. The left atrial posterior wall (LAPW) is also recognized as an important non-PV origin [6]. Structural remodeling and conduction abnormalities in this region may contribute to the maintenance and initiation of AF, particularly in patients with recurrent arrhythmia after PVI. However, there have rarely been previous reports describing the association between local bigeminal activity from LAPW and its arrhythmogenicity. Here, this case represents local bigeminal activity in the LAPW preceding AF initiation. The activity was detected adjacent to a low-voltage region, suggesting a potential interaction between localized electrical activity and diseased atrial substrate, with important implications for optimizing catheter ablation strategies in patients with AF.

## Case report

A 70-year-old woman underwent cryoballoon ablation for symptomatic paroxysmal AF. One month later, the patient revisited our hospital complaining of palpitations due to AF recurrence. Antiarrhythmic drugs, including a beta-blocker and bepridil, were administered, but paroxysmal AF was not improved 5 months after the initial procedure. Therefore, we performed a second session of ablation by radiofrequency catheter ablation (RFCA). Voltage mapping of the left atrium (LA) using an OCTARAY mapping catheter and the CARTO3 system (Biosense Webster, Irvine, CA, USA) revealed re-connection at the posterior carina of the right inferior PV (RIPV) as well as a low voltage area on the LAPW. After re-isolation of the RIPV, we performed atrial burst pacing and induced AF. Immediately after sinus rhythm recovery following electrical cardioversion (DC), localized bigeminal activity was recorded in the LAPW by the OCTARAY (Fig. 1). The coupling interval of the bigeminal activity ranged approximately from 130 to 150 ms with modest variability. Interestingly, the bigeminal potentials were only observed in a localized area (Fig. 2A, spline C/D) and were clearly distinct from the surrounding atrial electrical activities. Shortly after the appearance of this activity, AF was repeatedly initiated from the same region. AF spontaneously terminated within several minutes but recurred repeatedly from the LAPW following the localized bigeminal activity. Based on these findings, the LAPW was considered the potential initiating region for recurrent AF. Subsequently, targeted focal ablation by RFCA at the site of bigeminal activity resulted in transient AF termination, but AF recurred shortly thereafter. To achieve durable elimination, linear ablation connecting the site of bigeminal activity to the adjacent low-voltage region of LAPW was performed (Fig. 3). This intervention resulted in termination of AF and rendered the arrhythmia non-inducible under our institutional protocol. The patient has remained free from any arrhythmia recurrence during 1 year of follow-up after the second session.

**Fig. 1:**
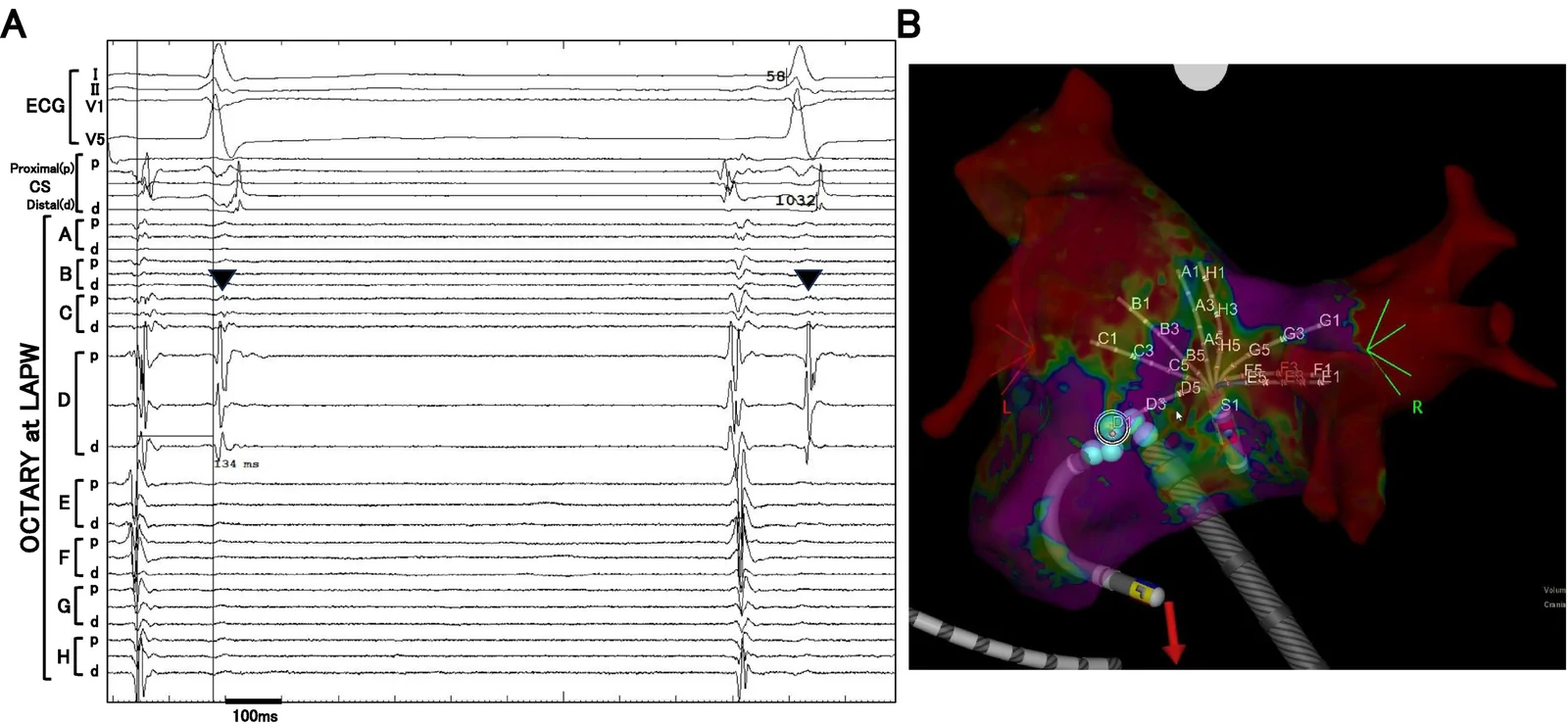
(A) Localized bigeminal activities from the LAPW. Arrow heads show localized premature electrical activities. (B) Voltage mapping of the LA. Blue points show fragmentated potentials. ECG, electrocardiogram; CS, coronary sinus; LAPW, left atrial posterior wall.

**Fig. 2:**
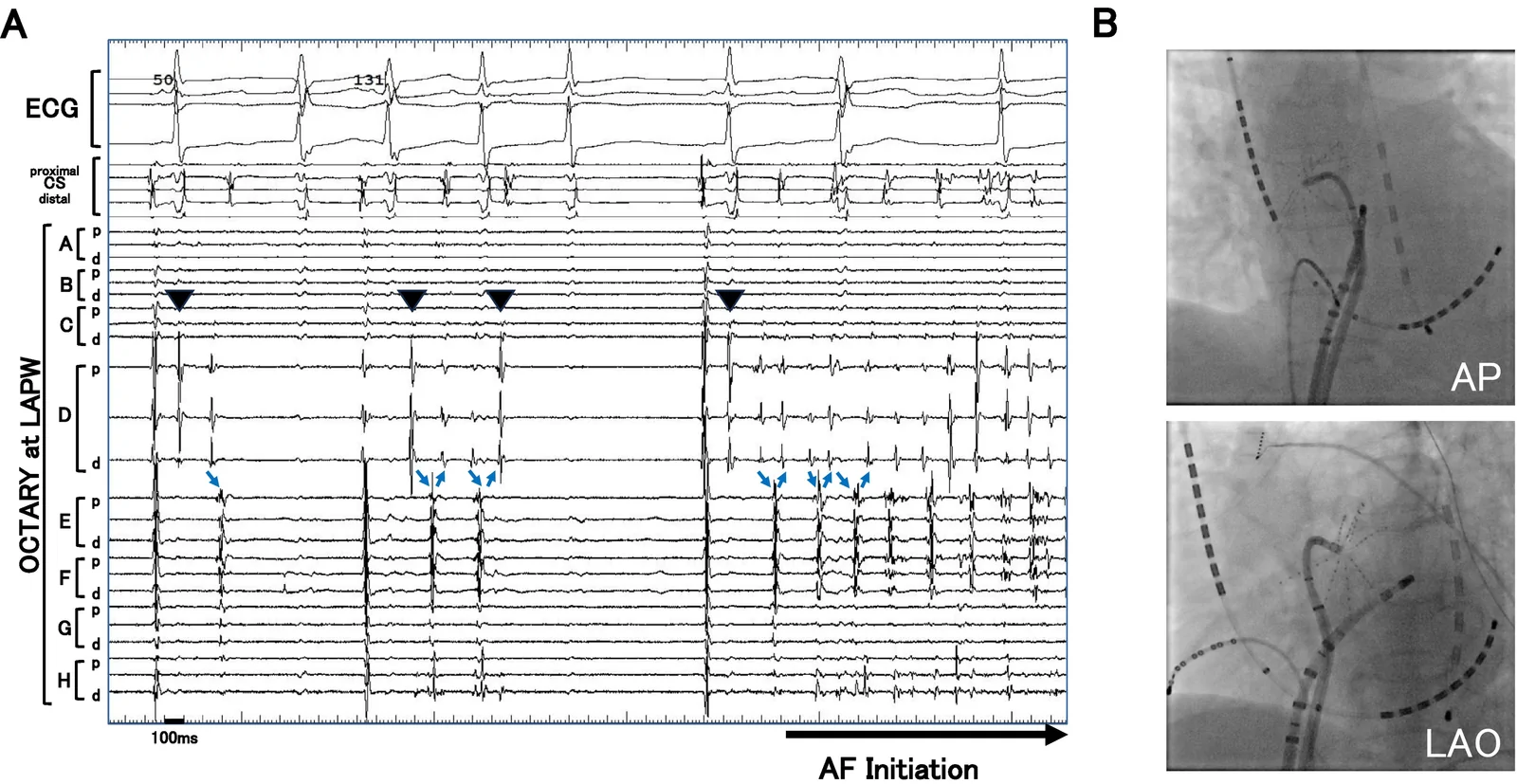
(A) AF was induced following the bigeminal activities. Blue arrows indicate electrical gradation in the adjacent splines. (B) Fluoroscopic images at the success point of RFCA. ECG, electrocardiogram; CS, coronary sinus; LAPW, left atrial posterior wall; AF, atrial fibrillation; AP, anterior-posterior view; LAO, left anterior oblique; RFCA, radiofrequency catheter ablation.

**Fig. 3:**
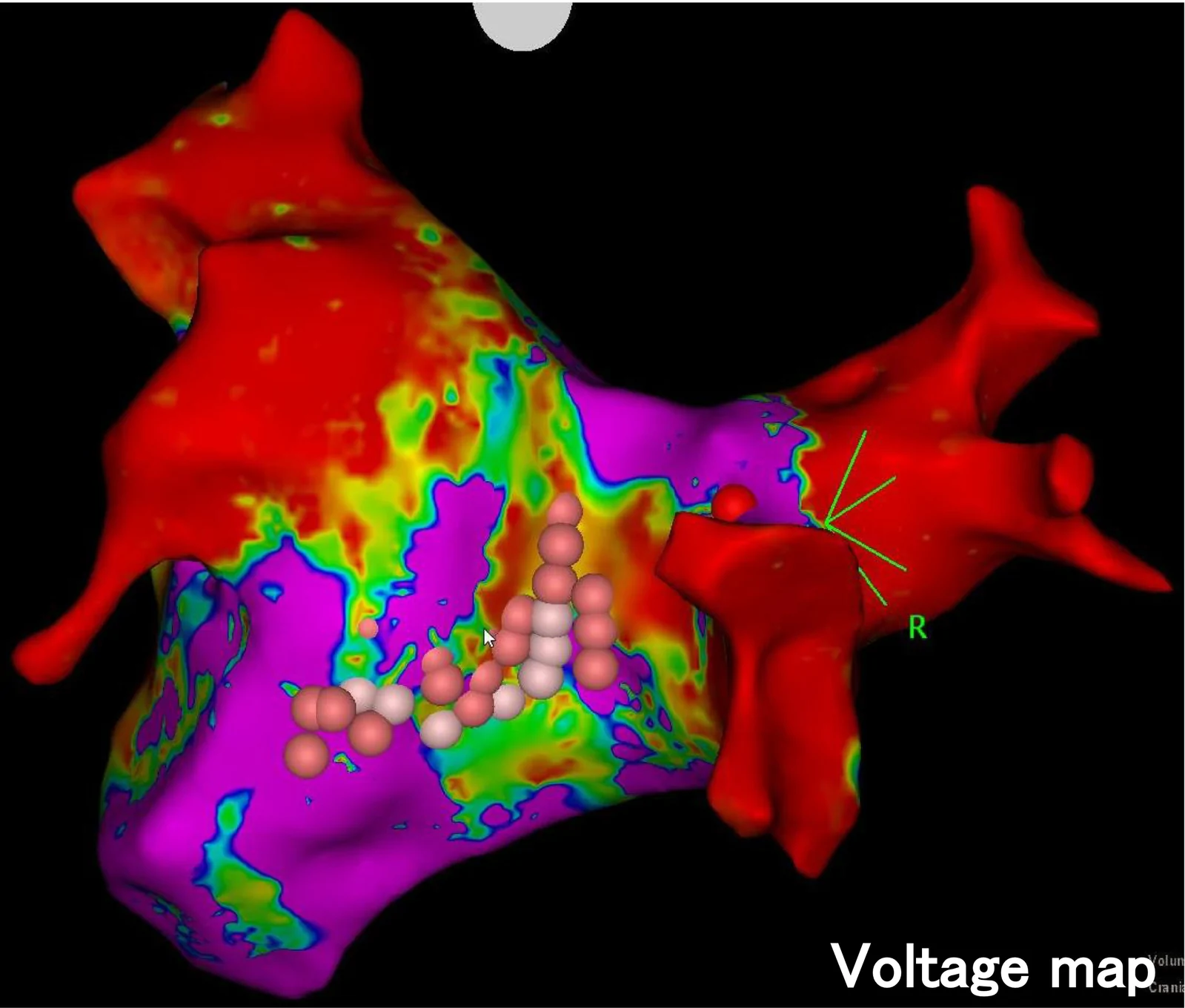
Representative images of the CARTO mapping. Voltage mapping and ablation points.

## Discussion

This case demonstrated localized bigeminal activity confined to a restricted region of LAPW preceding AF initiation. While bigeminal activity arising from the PVs [4] or the SVC [5] has previously been associated with arrhythmogenic potential, similar localized activity originating from the LAPW has rarely been described. In the present case, the bigeminal activity was observed adjacent to a low-voltage area of the LAPW. Although the earliest electrogram was recorded on spline D, which did not itself exhibit markedly reduced voltage, the neighboring splines (B and C) were located within the low-voltage area. This spatial relationship suggests that conduction abnormalities associated with structurally remodeled atrial myocardium may have contributed to the observed electrical activity. Notably, following the second component of the bigeminal activity, no clear endocardial propagation was observed in the surrounding electrodes. One possible explanation is that activation propagated through myocardial bundles located within or behind the low-voltage area, potentially involving epicardial myocardial fibers that were not directly captured by the endocardial electrodes. These findings suggest that localized electrical activity arising near a diseased atrial substrate may have contributed to the initiation of AF in this case.

Previous studies have reported that non-conducted ectopic discharges may represent a characteristic electrophysiological feature of arrhythmogenic triggers initiating AF [1], [7]. In the present case, although the amplitude of the bigeminal electrogram was relatively preserved, the second component of the activity did not clearly propagate to the surrounding atrial myocardium. This phenomenon may share electrophysiological similarities with non-conducted ectopic discharges described in previous reports. Considering that the activity was observed adjacent to a low-voltage area of the LAPW, the underlying mechanism may involve conduction abnormalities within structurally remodeled atrial tissue. One possible explanation is localized micro-reentrant activation or decremental conduction occurring around scarred or fibrotic myocardium, which may contribute to the initiation of AF. Mukai and colleagues reported that bigeminal potentials of PVs could be an indicator of AF arrhythmogenicity, and that the underlying mechanism may involve reentry within or around the PVs due to the presence of decremental conduction properties [4]. Several studies have emphasized the role of the LAPW as a substrate for reentry and rotors, particularly in the setting of persistent AF [6], [8]. It is also noted that the activation pattern of the bigeminy site (Fig. 2A; black arrowhead and blue arrow) showed electrical gradation in time phase, suggesting the hypothesis of reentrant circuit involving the different layers of the myocardium. The reentrant circuit might be involved in the mechanisms either through a low-voltage area adjacent to the site of bigeminal activity [3] or vein of Marshall (VOM). Mukai et al. also reported that PV bigeminy, especially in the left inferior PV, may be associated with epicardial conduction through VOM [4]. In our case the electrophysiological information on VOM is lacking and so the involvement of VOM remains speculative. Han and colleagues identified ectopic activities originating from VOM by inserting electrodes into VOM or an epicardium and evaluated electrical propagation to the LA via Marshall bundle [9]. In their report, multiple connections from the VOM to the coronary sinus or the LA wall were detected as fragmented potentials. In the present case, local fragmented potentials were detected at the sites of bigeminal activities, suggesting a locally propagating reentry pathway involving epicardial VOM. Another possibility is that the bigeminal activity represents triggered activity caused by afterdepolarizations, potentially facilitated by structural remodeling of the LAPW. In the present case, the coupling interval of the bigeminal activity showed modest variability, and the conduction pattern between adjacent electrodes was not entirely uniform. Rather than indicating incidental electrical activity, these findings may reflect heterogeneous conduction within structurally remodeled atrial myocardium. Such variable conduction patterns are frequently observed in regions with anisotropic conduction or partial conduction block. Although the precise electrophysiological mechanism cannot be definitively determined, the repeated observation of the bigeminal activity within a confined region of the LAPW immediately preceding AF initiation suggests that this region functioned as a localized initiating substrate. The response to ablation further supports this interpretation. Focal ablation at the site transiently suppressed AF but did not completely eliminate the arrhythmia. Durable elimination was achieved only after linear ablation connecting the site to the adjacent low-voltage area, suggesting the clinical implication that modification of the underlying substrate may have been necessary to eliminate the arrhythmogenic activity [6], [8].

In this report, there are several limitations. This is a single case, and the precise electrophysiological mechanism underlying the observed activity cannot be definitively established. Whether similar findings will be observed in other patients with recurrent AF after PVI remains to be determined.

We reported the case of AF initiated by bigeminal activity localized to the LAPW. Electrogram recordings demonstrated that the bigeminal activity was confined to a local area, distinct from surrounding atrial electrograms, contributed to AF initiation, and was spatially associated with an adjacent low-voltage substrate. These findings expand the current understanding of non-PV triggers and highlight the potential role of the LAPW as both a substrate and an initiating region in AF. Recognition of LAPW bigeminy may have important implications for ablation strategies and warrants further investigations.

## Consent statement

Written informed consent was obtained.

## Declaration of competing interest

We have no conflict of interest.
